# Unraveling persistent dyspnea after mild COVID: insights from a case series on hyperventilation provocation tests

**DOI:** 10.3389/fphys.2024.1394642

**Published:** 2024-07-26

**Authors:** Ophélie Ritter, Sofia Noureddine, Lucie Laurent, Pauline Roux, Virginie Westeel, Cindy Barnig

**Affiliations:** ^1^ Department of Chest Disease, University Hospital Besançon, Besançon, France; ^2^ Université de Franche-Comté, CHU Besançon, EFS, INSERM, UMR RIGHT, Besançon, France

**Keywords:** COVID-19, dyspnea, PETCO_2_, hyperventilation syndrome, hyperventilation provocation tests

## Abstract

Dyspnea is a common yet poorly understood symptom of long COVID, affecting many patients. This brief report examines the role of dysfunctional breathing in persistent dyspnea among patients with mild post-COVID-19 using hyperventilation provocation tests (HVPT). In this case series, six patients with unexplained dyspnea and normal cardiopulmonary function underwent HVPT. Despite normal exercise testing results, all patients exhibited delayed PETCO_2_ recovery, indicative of a hyperventilation pattern consistent with chronic hyperventilation syndrome, without typical symptomatic manifestations. These findings suggest underlying post-COVID respiratory dysregulation, emphasizing the importance of targeted diagnostic and therapeutic approaches for persistent respiratory symptoms in long COVID patients.

## Introduction

The term « long COVID » assembles a variety of long-term symptoms that persist or develop 3 months after a known or suspected SARS-CoV-2 infection, last for at least 2 months and cannot be explained by alternative diagnoses (WHO, 2024). Affecting up to 10%–20% of people infected by SARS-CoV-2 people, it represents nowadays a challenge for physicians as well as a social and economic burden.

Persistent dyspnea is notably prevalent among patients who initially experienced mild COVID-19 symptoms, lasting for months following the onset of the infection (Montani et al., 2022). Remarkably, this condition appears to be disproportionate, especially given that these patients typically demonstrate normal cardiopulmonary function upon extensive clinical evaluations. The underlying mechanisms driving the sustained experience of dyspnea remain poorly understood, although dysfunctional breathing with chronic hyperventilation has been proposed as a potential factor (Motiejunaite et al., 2021).

The hyperventilation provocation test (HVPT) has been designed to diagnose chronic hyperventilation syndrome. The test is typically proposed in patients with dyspnea associated to other symptoms of chronic hyperventilation including dizziness, paraesthesias, muscle stiffness, cold extremities, and trembling (Gardner et al., 1986). The test is considered positive when patients acknowledge that the symptoms induced during the test resemble those they encounter in their everyday lives. Additionally, the test assesses also the recovery kinetics of end-tidal carbon dioxide tension (PETCO_2_), which tend to differ in patients with chronic hyperventilation syndrome (HVS) (Vansteenkiste et al., 1991a).

## Case series

Here we report a case series of six patients from our clinic who were assessed for unexplained persistent dyspnoea (mMRC ≥ 1) following a mild course of COVID-19 infection. Out of all patients evaluated for persistent dyspnoea, those with obvious HVS, characterized by highly positive Nijmegen scores and/or clear dysfunctional breathing on CPET, were excluded from this series. This case series specifically includes patients with no apparent HVS.

Although their clinical symptoms and cardiopulmonary exercise testing results did not clearly indicate dysfunctional breathing, all six patients exhibited delayed recovery times in the PETCO_2_ after undergoing a hyperventilation provocation challenge. This was accompanied by an excessive, sustained minute ventilation due to a persistent yet asymptomatic increase in respiratory rate after the challenge.

The series comprised five women and one man, ranging in age from 27 to 54 years, with an average age of 38 years (±11.97) (Table 1). All patients experienced a mild course of COVID-19, without the need for hospitalization or oxygen therapy. On average, 8.2 months (±5.1) had elapsed since their last COVID-19 infection. None of the patients had a significant medical history, particularly concerning chronic cardiovascular or pulmonary conditions. The average BMI was 26.62 (±6.85), and all participants were non-smokers. Dyspnea severity varied, with mMRC scores ranging from 1 to 4. At the time of evaluation, all were sedentary, although their physical activity levels prior to contracting COVID-19 varied from sedentary to highly active. None reported the classical symptoms associated with chronic hyperventilation as dizziness, palpitations, numbness, and a tingling sensation. The Nijmegen Questionnaire, a symptom-based questionnaire to screen for hyperventilation syndrome (HVS) (van Dixhoorn and Duivenvoorden, 1985), exceeded marginally the threshold score of >23 in two patients, yielding an average score of 22 (±7.1). All participants demonstrated normal results in pulmonary function tests, with a mean forced expiratory volume in 1 s (FEV1) of 97.92% (±13.3) and a mean forced vital capacity (FVC) of 99.01% (±9.96) of the predicted values. Additionally, diffusing capacity of the lung for carbon monoxide (DLCO) was within normal limits. Cardiological evaluations and chest imaging for each patient also returned normal findings.

**TABLE 1:** Characteristics of patients presenting with persistent dyspnea in the aftermath of COVID-19, and findings of cardiopulmonary exercise testing and hyperventilation provocation tests.

	Case 1	Case 2	Case 3	Case 4	Case 5	Case 6	Mean ± SD
Main characteristics							
Gender	Female	Female	Female	Female	Female	Male	
Age, years	42	29	27	54	49	27	38 ± 11.97
BMI, kg/m^2^	32, 5	23, 9	17, 4	25, 2	36, 6	24, 1	26.62 ± 6.85
Time from last infection (months)	5	5	9	7	5	18	8.2 ± 5.1
Physical profile before COVID-19	Not active	Very active	Very active	Active	Not active	Active	
Dyspnoea mMRC	4	1	3	1	2	1	
Cardiopulmonary exercise testing							
Peak V′O_2_, mL/min/kg	15, 3	34, 3	42, 8	31, 1	23, 6	34	30, 18 ± 9, 55
Peak V′O_2,_ % pred	69	118	119	143	130	84	110.5 ± 28.2
WR reached, W	80	165	160	180	130	200	152.5 ± 42.4
% of predicted workload	75	115	137	182	130	77	42.4 ± 40.3
Peak RR (breath/min)	44	51	50	46	25	45	43.5 ± 9.5
Peak V′E/V′CO_2_	33	39	29	40	26	37	34 ± 5.66
Symptoms at exertion	Dyspnea, leg fatigue	Dyspnea	Dyspnea	Dyspnea, leg fatigue	Dyspnea, leg fatigue	Dyspnea	
Hyperventilation provocation test							
Adaptation phase (3 min)							
PETCO_2_ at start (mmHg)	36	36	37	31	31	37	35 ± 3.2
Mean PETCO_2_ (mmHg)	35.8 ± 0.7	36.8 ± 0.8	37.5 ± 0.5	30.3 ± 0.8	33.2 ± 1.4	36.7 ± 1.1	35.1 ± 2.7
Mean RR (br/min)	19.5 ± 1.9	9.4 ± 2,2	16 ± 1.1	12.8 ± 1.2	11.3 ± 1.6	15.14 ± 2.5	14 ± 3.6
Mean Vt (mL)	396.3 ± 89.8	1,068 ± 212.9	502.5 ± 47.5	769 ± 97.3	697.2 ± 97	792.3 ± 98.4	704.2 ± 236.6
Mean V′E (L/min)	7.6 ± 1	9.5 ± 1.5	7.9 ± 0.5	9.7 ± 0.9	7.8 ± 0.7	12 ± 1.9	9.1 ± 1.7
PETCO_2_ at end (mmHg)	36	36	38	32	35	34	35 ± 2.4
Voluntary hyperventilation phase (3 min)							
Minimum PETCO_2_ (mmHg)	14	13	12	11	15	15	13.3 ± 1.6
Fall PETCO_2_ (mmHg)	22	23	26	20	20	19	21.7 ± 2.6
Mean RR (br/min)	73.9 ± 22.1	52.3 ± 8.4	79 ± 6.4	69.5 ± 14.1	77.7 ± 12.8	77.8 ± 23.1	72.2 ± 10.6
Mean Vt (mL)	577.7 ± 119.1	1,209 ± 85.1	741.3 ± 28.3	1,016 ± 66.3	527.5 ± 128.9	781.3 ± 66.3	803.1 ± 263.3
Mean V′E (L/min)	44.1 ± 5	63.4 ± 11	58.7 ± 5.9	71 ± 16.9	41.1 ± 12.1	59.6 ± 13.7	56.2 ± 11.5
Recovery phase (3 min)							
PETCO_2_ at start	14	14	12	11	15	15	13.5 ± 1.6
PETCO_2_ 3 min (mmHg)	23	16	19	20	16	25	19.8 ± 3.6
△PETCO_2_ 3 min (mmHg) from start	9	2	7	9	1	10	6.5 ± 3.7
RR 3 min (br/min)	26	25	34	11	22	11	21.5 ± 9
Vt 3 min (mL)	406	880	414	777	1,492	776	790.8 ± 397.5
V′E 3 min (L/min)	10.5	22.2	14.3	8.3	32.9	8.9	16.2 ± 9.7
PETCO_2_ 5 min (mmHg)	24	18	20	24	16	26	21.3 ± 3.9
△PETCO_2_ 5 min (mmHg) from start	10	5	8	13	1	11	8 ± 4.4
RR 5 min (br/min)	26	15	34	18	18	17	21.3 ± 7.2
Vt 5 min (mL)	353	1,124	411	390	1,196	403	646.2 ± 399.2
V′E 5 min (L/min)	9.3	17	13.8	3.2	21.5	7	11.9 ± 6.7

Abbreviations: BMI, body mass index; FEV1, forced expiratory volume at first second; FVC, forced vital capacity; PETCO_2_, End-tidal CO_2_ tension; RR, respiratory rate; V′O_2_, oxygen uptake; V′E, minute ventilation; WR: work rate; V′E/V′CO_2_, ventilatory equivalents for CO_2_; VD, VT, tidal volume.

All individuals underwent maximal symptom-limited incremental cardiopulmonary exercise testing (CPET) on a cycle ergometer, which did not uncover any significant cardiorespiratory abnormalities (Table 1). Dyspnea and leg pain were the limiting factors. One participant had an aerobic capacity below 80% of the predicted value and did not meet the criteria for maximal effort due to lack of motivation. There were no clear observations of an excessive ventilatory responses to exercise with a mean peak respiratory rate (RR) of 43.5 breath/min (±9.5) and a mean minute ventilation to carbon dioxide output (V′E/V′CO_2_) of 34 (±5.7) and no erratic patterning of breathing frequency and/or tidal volume.

A HVPT was proposed to all patients as previously described (Vansteenkiste et al., 1991a). This test consisted of a 3 min baseline recording period of quiet breathing, during which the patient was asked to “breathe normally” (adaptation phase). This was followed by a 3-min phase of voluntary hyperventilation, during which patients were encouraged to significantly increase their tidal volume (VT) and respiratory rate (RR) as much as possible (voluntary hyperventilation phase). Following the hyperventilation phase, patients were asked to return to breathe normally for 5 min without specific instructions regarding their VT or RR (recovery phase). The PETCO_2_ and ventilation parameters were continuously monitored (Table 1). During hyperventilation phase, all participants achieved a RR > 50 breaths per minute and a minimum PETCO_2_ that fell than more than 50% compared to the rest PETCO_2._ Patients were also requested to report any symptoms experienced during the test.

All the patients had a baseline PETCO_2_ > 30 mmHg with a mean baseline PETCO_2_ at start of 35 ± 3.2 mmHg that remained stable during the adaptation phase. Profound hypocapnia (<20 mmHg) was obtained in all patients during the hyperventilation phase. Surprisingly, none of the patients experienced typical hypocapnia-related disabling symptoms. None of the subjects recovered basal PETCO_2_ before the end of the recovery phase with a mean PETCO_2_ of 19.8 ± 3.6 at the 3 min and of 21.3 ± 3.9 at the 5 min. The mean increase from the maximum drop PETCO_2_ was 6.5 ± 3.7 at the 3 min and 8 ± 4.4 at the 5 min. Throughout the entire recovery phase, all the patients maintained elevated VE accompanied by an increased RR with a mean RR of 21.3 ± 7.2 at the 5 min. Despite these significant physiological changes, the patients remained unaware of the alterations and reported no symptoms of dyspnea or any discomfort typically associated with hypocapnia.

## Discussion

Our case series focused on patients with unexplained dyspnea after mild COVID-19 who exhibited delayed recovery times in the PETCO_2_ after undergoing a hyperventilation provocation challenge, as typically observed during chronic HVS (Vansteenkiste et al., 1991a). Unlike in usual chronic HVS, our patients did not experience the typical symptoms of hypocapnia commonly associated with chronic HVS but complained mainly of mild yet uncomfortable persistent exercise-induced dyspnea. This was further evidenced by mostly negative results on the Nijmegen Questionnaire, a widely used instrument based on a symptom-based questionnaire for identifying individuals with chronic HVS (van Dixhoorn and Duivenvoorden, 1985). This discrepancy suggests that while the Nijmegen score is a valuable tool, it may not capture all forms of dysfunctional breathing, especially in post-COVID-19 patients. Indeed, in later studies, the correlation between Nijmegen Questionnaire scores and carbon dioxide tensions appeared highly variable, and its lack of diagnostic accuracy has been acknowledged, making it more suitable for monitoring the evolution of symptoms rather than for making a diagnosis of chronic HVS (van Dixhoorn and Folgering, 2015).

Chronic HVS occurs predominately at rest but can occur during exercise (Howell, 1997). Cardiopulmonary exercise testing is thus regarded as a highly sensitive method for diagnosing chronic HVS, particularly when exertional dyspnea is the predominant symptom (Warburton and Jack, 2006). Patients with hyperventilation syndrome (HVS) typically tolerate lower exercise workloads and exhibit abnormal breathing patterns during physical activity, compared to healthy individuals (Chenivesse et al., 2014). However, this characteristic was not observed in our case series.

In the HVPT, individuals with chronic HVS typically begin with a PETCO_2_ level below 30 mmHg, a trait not seen in our patients (Vansteenkiste et al., 1991b). Furthermore, those with chronic HVS often experience a decrease in PETCO_2_ levels during the adaptation phase, a pattern that did not occur in our patient group (Vansteenkiste et al., 1991b).

Similar to typical chronic HVS, all our patients exhibited atypical PETCO_2_ recovery kinetics following the HVPT, with none achieving their baseline PETCO_2_ levels by the end of the recovery phase. The phenomenon of delayed PETCO_2_ normalization after voluntary hyperventilation in chronic HVS patients, has been described for a long (Grossman and de Swart, 1984).

Different diagnostic thresholds have been proposed, such as a PETCO_2_ value below 67% of the baseline at the 3 min of recovery and/or below 91% at the 5 min (Beumer and Hardonk, 1971; Vansteenkiste et al., 1991b). A more recent criterion identifies a rise of less than 12.8 mmHg in PETCO_2_ at the 5 min of recovery as indicative of hyperventilation syndrome, with a high sensitivity (0.92) and specificity (0.84) (Pauwen et al., 2022). All our patients met the criteria, regardless of the specific threshold applied. Similar to patterns observed in hyperventilation syndrome, our patients exhibited elevated VE during the recovery phase, primarily due to an increased RR. Remarkably, despite the persistent tachypnea, none of the patients reported discomfort or experienced sensations of dyspnea during the recovery period.

There is emerging literature suggesting that dysfunctional breathing should be considered in many patients with persistent and unexplained dyspnea after a COVID infection (Motiejunaite et al., 2021; Beurnier et al., 2023; Genecand et al., 2023). Our patients were predominantly female which is in concordance with data showing the female sex is a risk factor for persisting symptoms after SARS-CoV-2 infection (Subramanian et al., 2022). The origin of this disabling dysfunctional breathing following mild COVID infection is unknown. Consistent with chronic hyperventilation syndrome, psychological and behavioral contributors might be implicated. Banzett demonstrated that dyspnea engages neural pathways shared with pain and is influenced by similar psychological and emotional factors, particularly in the insular cortex and limbic structures (Lansing et al., 2009). In a specific study focusing on individuals with ongoing functional respiratory complaints post-COVID infection, selection for a HVPT was based on a positive Nijmegen test, which showed an average score of 31.21 ± 5.05 (Beurnier et al., 2023). In the study, a significant correlation was found between the Nijmegen scores and the presence of anxiety and depression symptoms. During the HVPT, abnormal reactions were observed in 21 out of 25 patients (84%). Unlike our patient group, these individuals developed their usual daily symptoms with major discomfort, which often resulted in the premature interruption of the hyperventilation challenge. However, our patients presented a distinct clinical profile, with persistent dyspnea as their predominant symptom, lower Nijmegen scores, and a lack of the usual functional respiratory symptoms during the HVPT, despite experiencing significant hypocapnia. In the other hand, considering that the angiotensin-converting enzyme 2 (ACE2), the receptor for the COVID-19 virus, is present in brainstem nuclei that regulate ventilation (Baig et al., 2020), the hyperventilation observed may also stem from an organic origin linked to post-COVID-19 changes, potentially involving disruptions in central respiratory control. Possible mechanisms may involve either an overactivity of stimulatory systems or a failure of inhibitory pathways. The difficulty of our patients to retrieve a normal resting VE after a hyperventilation challenge might also indicate an unusual hyposensitivity to low CO_2_ levels, a phenomenon suggested already four decades ago in chronic hyperventilation syndrome (Folgering and Durlinger, 1983). This underscores the need for further investigation into the underlying mechanisms.

Finally, chronic HVS can significantly impair the quality of life in affected individuals. While there is no standardized treatment, conventional approaches typically involve physiotherapy, patient education and breathing retraining. The efficacy of these treatments, however, lacks strong evidence (Jones et al., 2013). There is growing awareness that dyspnea is a multidimensional experience. According to research by Banzett et al. (2015) this experience encompasses sensory, affective, and cognitive dimensions, which interact to shape the overall perception of dyspnea. The Multidimensional Dyspnea Profile (MDP), is an instrument developed by Banzett and colleagues to assess these dimensions (Banzett et al., 2015). Although it has not yet been tested specifically for patients with persistent dyspnea after mild COVID-19, it has the potential to identify unique patterns and characteristics of dyspnea in post-COVID syndrome. By capturing the nuanced experiences of dyspnea through the MDP, physicians can gain a comprehensive understanding of the specific challenges faced by these patients. This detailed assessment could facilitate more personalized and effective treatment strategies (Banzett et al., 2015). In our case series, patients underwent breathing retraining and exercise, which resulted in some improvement but did not fully resolve the symptoms.

## Conclusion

In conclusion, our case series highlights the complexity of persistent dyspnea in post-mild COVID-19 patients, underscoring the potential role of dysfunctional breathing and the diagnostic value of HVPT. Despite normal cardiopulmonary function and the absence of typical HVS symptoms, patients exhibited significant alterations of PETCO_2_ kinetics and ventilation patterns after the hyperventilation challenge, suggesting an underlying dysregulation potentially exacerbated by COVID-19. These findings call for a deeper understanding of post-COVID respiratory issues and emphasize the need for tailored therapeutic approaches to address the unique challenges of long COVID.

## Data Availability

The raw data supporting the conclusions of this article will be made available by the authors, without undue reservation.
